# Prevalence of and risk factors for post-intensive care syndrome: Multicenter study of patients living at home after treatment in 12 Japanese intensive care units, SMAP-HoPe study

**DOI:** 10.1371/journal.pone.0252167

**Published:** 2021-05-27

**Authors:** Takeshi Unoki, Hideaki Sakuramoto, Sakura Uemura, Takahiro Tsujimoto, Takako Yamaguchi, Yuko Shiba, Mayumi Hino, Tomoki Kuribara, Yuko Fukuda, Takumi Nagao, Mio Kitayama, Masako Shirasaka, Junpei Haruna, Yosuke Satoi, Yoshiki Masuda, on behalf of the SMAP-HoPe Study Project

**Affiliations:** 1 Department of Acute and Critical Care Nursing, School of Nursing, Sapporo City University, Sapporo, Hokkaido, Japan; 2 Department of Adult Health Nursing, College of Nursing, Ibaraki Christian University, Hitachi, Ibaraki, Japan; 3 Emergency and Critical Care Medical Center, Osaka City General Hospital, Osaka, Japan; 4 Nursing Practice and Career Support Center, Nara Medical University Hospital, Kashihara City, Nara, Japan; 5 Intensive Care Unit, Nippon Medical School Musashikosugi Hospital, Kawasaki, Kanagawa, Japan; 6 Intensive Care Unit, University of Tsukuba Hospital, Tsukuba, Ibaraki, Japan; 7 Intensive Care Unit, Tohoku Medical and Pharmaceutical University Hospital, Sendai, Miyagi, Japan; 8 Intensive Care Unit of Advanced Emergency Medical Service Center, Japanese Red Cross Maebashi Hospital, Maebashi, Gunma, Japan; 9 Intensive Care Unit, Jichi Medical University Hospital, Yakushiji Shimotsuke-shi, Tochigi, Japan; 10 Intensive Care Unit, Sakakibara Heart Institute, Fuchu-shi, Tokyo, Japan; 11 Nursing Department Heart Center, Kanazawa Medical University Hospital, Uchinada, Ishikawa, Japan; 12 Intensive Care Unit & Cardiac Care Unit, Japanese Red Cross Fukuoka Hospital, Fukuoka, Japan; 13 Intensive Care Unit, Sapporo Medical University Hospital, Sapporo, Hokkaido, Japan; 14 Intensive Care Unit, Naha City Hospital, Naha, Okinawa, Japan; 15 Department of Intensive Care Medicine, Sapporo Medical University Hospital, Sapporo, Hokkaido, Japan; Rutland Regional Medical Center, UNITED STATES

## Abstract

Few studies have examined the epidemiology of post-intensive care syndrome in Japan. This study investigated the mental health and quality of life of patients living at home in Japan after intensive care unit (ICU) discharge. Additionally, we examined whether unplanned admission to the ICU was associated with more severe post-traumatic stress disorder (PTSD), anxiety, and depressive symptoms. An ambidirectional cohort study was conducted at 12 ICUs in Japan. Patients who stayed in the ICU for > 3 nights and were living at home for 1 year afterward were included. One year after ICU discharge, we retrospectively screened patients and performed a mail survey on a monthly basis, including the Impact of Event Scale—Revised (IER-S), the Hospital Anxiety Depression Scale (HADS), and the EuroQOL—5 Dimension (EQ-5D-L) questionnaires. Patients’ characteristics, delirium and coma status, drugs used, and ICU and hospital length of stay were assessed from medical records. Descriptive statistics and multilevel linear regression modeling were used to examine our hypothesis. Among 7,030 discharged patients, 854 patients were surveyed by mail. Of these, 778 patients responded (response rate = 91.1%). The data from 754 patients were analyzed. The median IES-R score was 3 (interquartile range [IQR] = 1‒9), and the prevalence of suspected PTSD was 6.0%. The median HADS anxiety score was 4.00 (IQR = 1.17‒6.00), and the prevalence of anxiety was 16.6%. The median HADS depression score was 5 (IQR = 2‒8), and the prevalence of depression was 28.1%. EQ-5D-L scores were lower in our participants than in the sex- and age-matched Japanese population. Unplanned admission was an independent risk factor for more severe PTSD, anxiety, and depressive symptoms. Approximately one-third of patients in the general ICU population experienced mental health issues one year after ICU discharge. Unplanned admission was an independent predictor for more severe PTSD symptoms.

## Introduction

Many patients, once discharged from intensive care units (ICUs) and living at home, experience discomfort and struggle with memories of being in the ICU [1]. It is widely recognized that ICU survivors experience several symptoms post-discharge, including mental health-related disorders, impaired cognitive and physical function, and decreased quality of life (QOL) [2]. These new or worsened impairments during and after intensive care are known as post-intensive care syndrome (PICS) [3]. PICS consists of psychological, physical, and cognitive impairments [3]. In particular, psychological impairments post-ICU are associated with perceived unacceptable outcomes [4] and decreased QOL [5]. A previous study conducted in the UK reported that 18%, 38%, and 32% of patients had post-traumatic stress disorder (PTSD), anxiety, and depression, respectively—even one year after being discharged from an ICU [6]. Additionally, a previous meta-analysis indicated that 19.8% of patients had significant PTSD after an ICU stay [7].

Several studies reported the prevalence of mental health issues, such as PTSD [7], after discharge from the ICU; however, most of these studies were conducted in Europe and the US. Little is known about the situation in Asia, including in Japan [7]. Because the prevalence of mental health issues vary across countries [8], it is worth investigating its incidence in various countries and regions. Recently, Wu and colleagues [9] evaluated the prevalence of mental health issues in post-ICU patients in Hong Kong. In Japan, in a recent single-center cohort study, Shima and colleagues [10] found that 3 and 12 months after ICU discharge, over half of the Japanese patients admitted to the emergency unit experienced impaired activities of daily living and/or psychiatric symptoms. They reported that the prevalence of PTSD was 20% at 12 months post-ICU discharge. Although this prevalence was consistent with that reported in a previous systematic review [7], the population did not reflect the general ICU population in Japan. Kawakami and colleagues [11] reported the effects on physical and mental QOL, as measured by the Short Form 36, and cognitive impairment after 6 months among 96 patients who required mechanical ventilation (MV) for more than 48 hours in 16 ICUs in Japan. However, they did not clarify the prevalence of PTSD, anxiety, or depression.

It is also essential to understand the determinants of PICS so that preventive measures such as multidisciplinary interventions [12] can be tailored to suit each survivor [13]. As far as we know, the mechanism behind PTSD is not fully understood. Patients’ memories are one possible factor for the occurrence of PTSD. A considerable number of ICU patients have delusional memories, such as dreams, nightmares, and paranoid delusions, as well as hallucinations [14]. A recent study [15] suggested that emotional memories may play a key role in PTSD development. Many studies [16–18] have attempted to clarify the risk factors for the development of PTSD after ICU stay. A recent systematic review and meta-analysis suggested that younger age, female patients, presence of delirium in the ICU, preexistence of a psychological disorder, administration of analgesics, and negative ICU experience were significant risk factors for the development of PTSD after ICU stay [19]. However, the role of the type of admission, such as unplanned admissions, in the development of PTSD after ICU care remains unclear. By understanding the determinants of PTSD, we can predict which patients are at a high risk of developing PTSD after discharge and ensure effective follow-ups.

This study aimed to clarify the prevalence of mental health issues, including PTSD, anxiety, and depression, as well as the QOL of Japanese ICU survivors who were living at home one year after discharge in a multicenter study. The secondary objective was to explore the risk factors associated with mental health impairment. We hypothesized that unscheduled admission to the ICU would be associated with severity of PTSD, anxiety, and depression symptoms as a surrogate for PICS in a mixed ICU population.

## Materials and methods

### Study design and setting

A multicenter ambidirectional cohort study was conducted at 12 ICUs (S1 Text), including 10 medical–surgical ICUs, one medical–surgical ICU that also specializes in emergency medicine, and one cardiovascular surgery ICU in different hospitals in various areas of Japan. This study was conducted from October 2019 to July 2020. ICUs sequentially participated after we received ethical approval from each institution. The characteristics of each institution and ICU are shown in S1 Table.

### Participants and recruitment process

We retrospectively enrolled consecutive participants who were discharged from the ICU 12 months earlier based on medical records at the time of hospital admission and prospectively surveyed their present health status using mail surveys. S1 Table shows that PICS measures at each ICU. None of the participating ICUs implemented an outpatient follow-up. To be eligible for inclusion, patients must have stayed in the ICU for at least three nights and have lived at home for one year since ICU discharge. Patients who had abnormal central nervous system function (determined based on diagnostic imaging), such as stroke, traumatic brain injury, and cerebral tumors, were excluded. Additionally, patients with severe cognitive impairment, readmission to the ICU within 12 months, or direct transfer to another hospital during ICU stay were excluded. Furthermore, we excluded patients who could not complete a self-administered questionnaire, could not be contacted by telephone, or who refused to participate in the survey.

**Fig 1:**
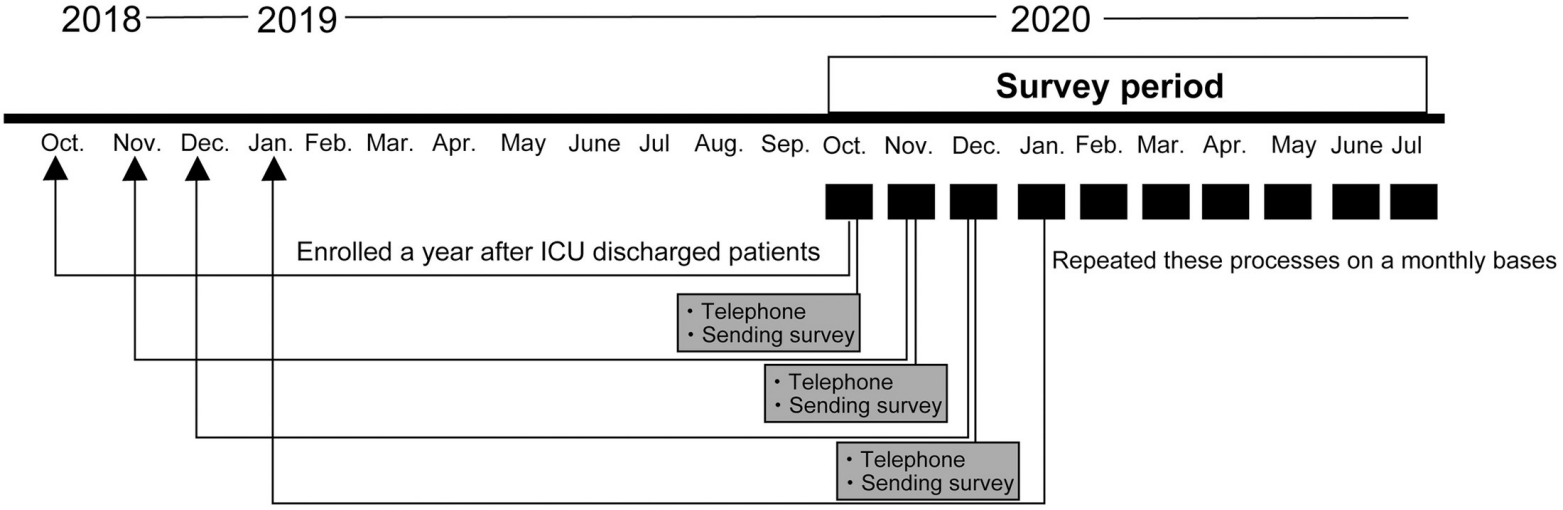
Schematic illustration of the study design. Each month, patients who had been discharged from ICU one year earlier were screened and contacted by telephone; thereafter, a survey set was mailed to these patients. ICU, intensive care unit.

The recruitment process is shown in Fig 1. Patients were consecutively screened on a monthly basis based on their medical records. Next, we mailed a letter to these patients, stating that a research nurse would telephone them within a few days using the address registered in the medical chart. A few days after sending the letter, the research nurse telephoned patients to confirm that they did not meet the exclusion criteria. At this time, we did not obtain formal informed consent but gave the patients a simple explanation of the study. Patients who refused to receive the survey were excluded. If the patients or their next of kin did not respond to at least three attempts of telephonic contact on different days, we recorded them as being “unable to contact.” After we confirmed which patients did not meet the exclusion criteria, a survey set, including an explanatory leaflet form and relevant questionnaires, was mailed. If a response was not received two weeks after sending the survey, a reminder was sent.

### Data collection

The survey set included the Impact of Event Scale-Revised (IES-R) [20], Hospital Anxiety and Depression Scale (HADS) [21] and EuroQOL—5 Dimension (EQ-5D-L) [22] questionnaires. Data concerning patients’ characteristics, delirium during ICU stay, and hospital outcomes were collected retrospectively from participants’ medical records.

All comorbidities were extracted from participants’ medical records. The Acute Physiology and Chronic Health Evaluation II (APACHE II) and Sequential Organ Failure Assessment were calculated using data obtained within 24 hours from ICU admission. Sepsis was defined according to the Sepsis-3 definition [23]. Exposure to benzodiazepines was defined as at least 24 hours of continuous intravenous infusion. Psychiatric history was defined as any record of psychiatric illness before ICU admission.

At all participating ICUs, nurses routinely assessed delirium at the bedside at least twice a day using the Confusion Assessment Method for the ICU [24], the Intensive Care Delirium Screening Checklist [25] or both. Similarly, the Richmond Agitation‒Sedation Scale [26] was assessed routinely in all ICUs. We defined the existence of delirium for at least one day as delirium, and if all assessed Richmond Agitation‒Sedation Scale scores were less than -3, we defined it as coma. The number of days of delirium and coma was measured during ICU stay. If we encountered mixed delirium and coma in 1 day, we defined it as delirium. These data were extracted from medical records.

#### Mental status

We used the Japanese version of the IES-R [27] and HADS [28] without modification to assess mental status. The IES-R is widely used to assess PTSD. The IES-R is a 22-item scale that measures how distressing each item was during the past week. It is rated from 0 (*not at all*) to 4 (*extremely*). The scale has three subscales: intrusion, avoidance, and hyper-arousal. The Japanese-translated version, which has been evaluated among different populations, has sensitivity and specificity values that range 0.75–0.89 and 0.71–0.93, respectively, at a cut-off of 25 for partial PTSD diagnosis [27]. We used this cut-off to define significantly symptomatic participants in this study.

The HADS has also been widely used and is a valid and reliable questionnaire for evaluating the degree of anxiety and depressive symptoms in an outpatient population [21] and critically ill patients [29]. It was translated into Japanese, which also has good reliability and validity [28]. The HADS consists of anxiety and depression subscales, and each subscale has seven items, which are rated on a scale from 0 to 3, with a total score ranging from 0 to 21. Half of the items relate to anxiety symptoms, and the other half relates to depressive symptoms. High correlations have been reported between the HADS score and diagnoses of anxiety and depression based on psychiatrist interviews (Spearman’s correlations: r = 0.70 and r = 0.74 for the severity of anxiety and depression, respectively) [28]. A score of ≥ 8 in each domain was defined as substantial anxiety or depression in the Japanese version of the HADS [28].

#### Quality of life

The EQ-5D-5L is a validated and standardized instrument used to measure health-related QOL [22]. We used a Japanese version of the EQ-5D-5L, without modification, which is available on request to the EuroQOL group (https://euroqol.org/). The EQ-5D-5L comprises five dimensions: mobility, self-care, usual activities, pain/discomfort, and anxiety/depression. Each dimension has five levels: no problem, slight problems, moderate problems, severe problems, and extreme problems. Health status is represented in 3,125 combinations, and each combination of answers can be converted into a QOL score, ranging from 0 (*death*) to 1 (*perfect health*) based on a Japanese value set [30]. We compared the score from our study with a previously reported Japanese norm [31]. The EQ-5D-5L also uses a visual analog scale (VAS), ranging from 0 to 100: 0 (*the worst imaginable health*) and 100 (*the best imaginable health*).

### Statistical analysis

The sample size calculation was based on a previous meta-analysis [7] that indicated that the prevalence of PTSD in ICU survivors was 19.8%. When we set the 95% confidence interval (CI) as less than ± 3%, the calculation revealed that 701 responses were needed. Additionally, for a multivariable linear regression model, when we set the number of dependent variables as 10, Cohen’s f^2^ as 0.025, and significance at.05, the calculation indicated that we needed 658 responses to reach 80% power. Therefore, we set the sample size at 700 responses.

For the analysis, descriptive statistics were derived. Continuous or ordinal data were expressed as medians with interquartile ranges (IQRs), unless otherwise specified. Data normality was tested visually and with the Shapiro-Wilk’s test. Categorical data were expressed as numbers, percentages, and 95% CIs. We used Fisher’s exact test to compare two or more categorical variables and the Kruskal‒Wallis rank test for comparisons of two variables with continuous or ordinal data.

We described variables for complete cases, except for the HADS and IES-R, unless otherwise specified. Missing items in the HADS and IES-R were imputed using the “half rule”: when half of the items in a subscale had responses, the mean of the responded score was imputed [32]. The HADS and IES-R scores were essentially representative after this imputation method.

We also used a Venn diagram to evaluate overlapping symptoms. Additionally, a sensitivity analysis was conducted after excluding trauma patients because this trauma was not ICU-related; rather, it involved events such as traffic accidents [33]. Subgroup analyses were performed between elective surgery and unplanned admission for characteristics and outcome data.

For the missing IES-R and HADS data that were not imputed by the “half rule” [32] because of many missing items, we additionally attempted to perform multiple imputations by chained equations (MICE) to avoid selection bias [34]. MICE was performed for individual total IES-R and HADS subscales. The imputed data were only used in multivariable analyses. Because the IES-R data were not normally distributed, we added + 1 to the sum of individual IES-R scores (to avoid infinity upon transformation); then, we transformed them to the natural logarithm for analysis.

We focused on the spectrum of PTSD, anxiety, and depression-related symptoms, rather than its diagnosis. To evaluate our hypothesis, we used a multilevel linear regression model to explore independent factors contributing to outcome variables. For PTSD symptoms, we chose covariates according to a conceptual model based on previous literature [35, 36] and clinical experience. Age, sex, number of days with delirium during ICU stay, duration of MV, ICU length of stay (LOS), use of benzodiazepines, and psychiatric history were selected as covariates and were adjusted for in the multivariable analysis. The duration of MV was selected because it could be reflected by the duration of receiving sedatives. Similarly, our hypothesis for anxiety and depression measured by HADS was evaluated using a multilevel linear regression model. We chose covariates according to a conceptual model based on previous literature [37, 38] and clinical perspective. In addition to age, sex, number of days with delirium during ICU stay, duration of MV, use of benzodiazepines, and psychiatric history, presence of malignancy was selected as a covariate when we analyzed anxiety and depression.

We used two-sided significance tests for all analyses, with significance set at p < .05. Analyses were performed in Stata 16.1 (StataCorp, College Station, TX).

### Ethical considerations

Ethical approval for this study was obtained by the main institution (Sapporo City University, Sapporo, Hokkaido, Japan, approval number: 1927–1). Additionally, ethical approval was obtained from the ethical committees of all participating institutions. We sent explanatory documents and consent forms with each survey set to the included patients. Participants were instructed to check a box on the consent form, which verified that they understood the research explanation and agreed to participate. In three institutions, consent was verified by patients writing their name on the form, according to each institutional review board’s recommendation.

## Results

**Fig 2:**
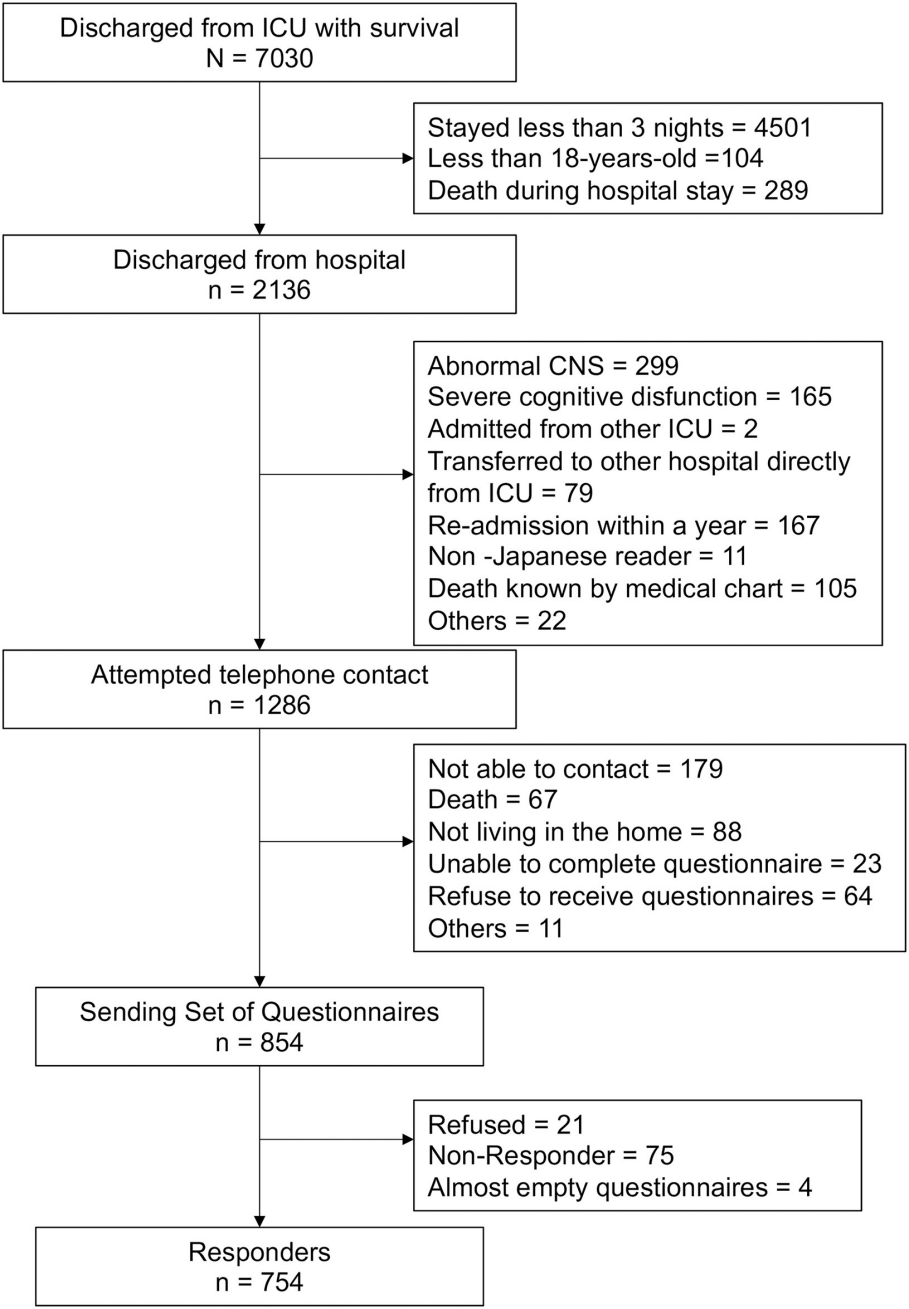
Patient recruitment flowchart. ICU, intensive care unit; CNS, central nervous system.

Fig 2 displays a flow diagram of study enrollment. We sent a survey set to 854 enrolled patients. Of these, 75 patients did not respond, and 778 patients returned the survey (response rate = 91.1%). Of the 778 participants who returned the survey, 21 patients refused participation, and four patients were excluded because their questionnaires were incomplete. These four patients were treated as non-responders. Consequently, the data from 754 (88.2%) participants were analyzed. Details of the missing variables in the IES-R and HADS are shown in S2 Table.

For the IES-R, 61 of 754 participants (8.1%) had missing data. After imputation for missing values in the IES-R using the “half rule” as described above, 36 (4.8%) missing data points remained; these missing scores were imputed with multiple imputation for multivariable analysis. Similarly, for the HADS, 66 (8.8%) patients had missing data; however, all HADS scores were imputed by the “half rule,” and we did not need to perform multiple imputation. Three participants (0.4%) had missing data for the QOL score, and 8 (1.1%) participants had missing data for the VAS in the EQ-5D-5L.

Table 1 shows the clinical characteristics of patients who did and did not respond to this study. The median ages of patients who did and did not respond to the survey were 70.0 (IQR = 61.0–78.0) and 70.0 (IQR = 49.5–79.0) years, respectively. Median APACHE II scores were 14.0 (IQR = 10.0–20.0) and 14.0 (IQR = 6.0–17.0), respectively. Median delirium (days), MV days, and ICU LOS were not significantly different between responders and non-responders. There was no significant difference between responders and non-responders, except for the proportion of the type of admission. Elective surgery, rather than unscheduled surgery, was more frequently the reason for admission in responders. In the 754 participants whose data were included, 358 (47.5%) were in the ICU for elective surgery.

**Table 1:** Demographics of responders and non-responders.

Variables		Responders n = 754	Non-responders n = 79	p
**Age (years), median [IQR]**		70.0 [61.0–78.0]	70.0 [49.5–79.0]	.43
**Female, n (%)**		213 (28.2)	29 (36.7)	.15
**Type of admission, n (%)**	**Elective surgery**	351 (46.6)	24 (30.4)	< .01
	**Unscheduled admission**	403 (53.4)	55 (69.6)	< .01
	**Unscheduled surgery**	108 (14.3)	19 (24.1)	.034
**Reason for ICU admission, n (%)**	**CV surgery**	307 (40.7)	27 (34.2)	.37
	**CHF/AMI/Arrhy**	121 (16.0)	14 (17.7)	
	**Sepsis**	78 (10.3)	13 (16.5)	
	**Abdominal surgery**	67 (8.9)	6 (7.6)	
	**ENT surgery**	31 (4.1)	0 (0.0)	
	**Respiratory failure**	30 (4.0)	5 (6.3)	
	**Aortic dissection (non-operative)**	26 (3.4)	4 (5.1)	
	**Other surgery**	25 (3.3)	3 (3.8)	
	**Trauma**	23 (3.1)	4 (5.1)	
	**Others**	46 (6.1)	3 (3.8)	
**APACHE II, median [IQR]**		14.0 [10–20]	14.0 [10.0–21.0]	.781
**MV use, n (%)**		518 (68.7)	50 (63.3)	.392
**MV (days), median [IQR]**		2 [0–3]	2 [0–2]	.377
**Benzodiazepines, median [IQR]**		49 (6.5)	5 (6.3)	1.0
**Psychological history, n (%)**		20 (2.7)	4 (5.1)	.387
**Delirium (days), median [IQR]**		0 [0–1]	0 [0–1.5]	.738
**ICU LOS (days), median [IQR]**		5 [4–7]	5 [4–6]	.295
**Hospital LOS (days), median [IQR]**		27.00 [19.00, 41.00]	31.00 [20.00, 43.50]	.539

IQR, interquartile range; CV, cardiovascular; CHF/AMI/Arrhy, congestive heart failure/acute myocardial infarction/arrhythmia; ENT, ear nose throat; APACHE II, Acute Physiology and Chronic Health Evaluation II; MV, mechanical ventilation; ICU, intensive care unit; LOS, length of stay.

### PTSD symptoms

**Fig 3:**
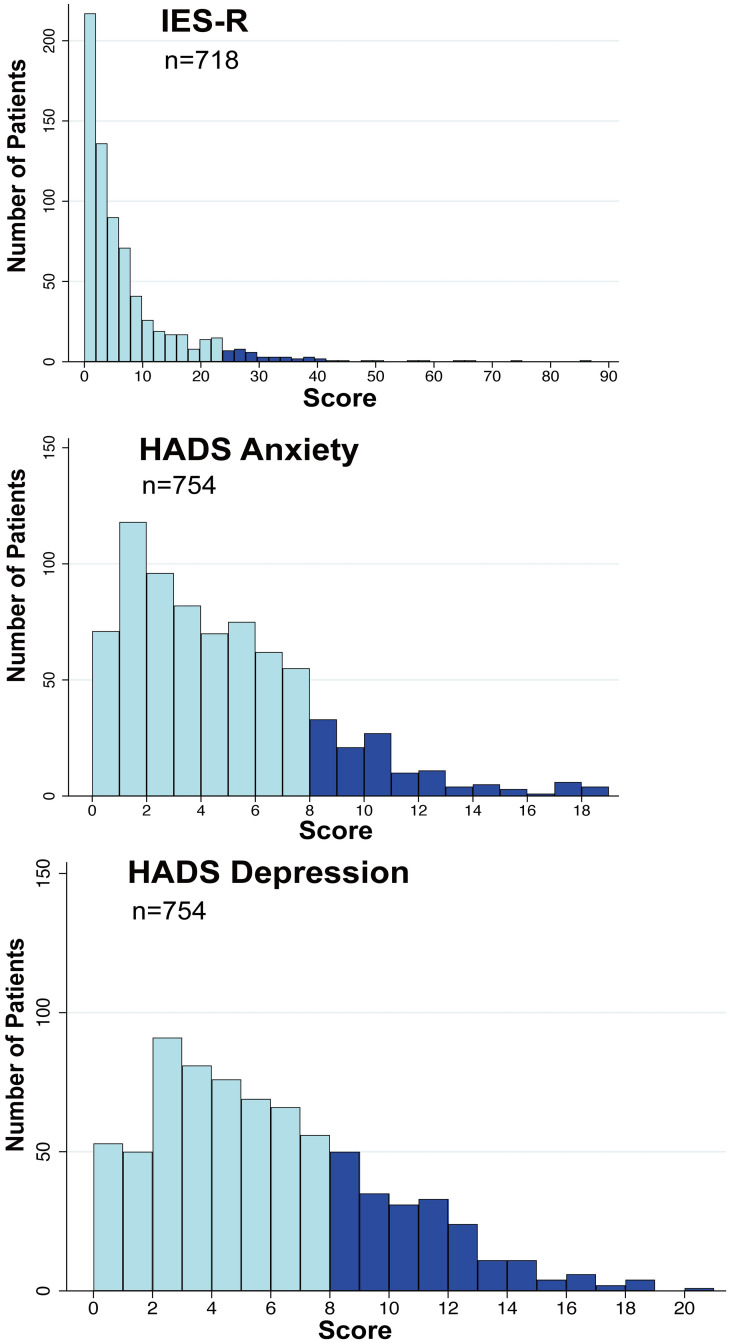
Histograms for IES-R and HADS scores in patients living at home one year after intensive care unit discharge. IES-R, Impact of Event Scale-Revised; HADS, Hospital Anxiety and Depression Scale.

The median total IES-R score was 3 (IQR = 1‒9). The prevalence of suspected PTSD (total IES-R > 24 points) was 6.0% (95% CI: 4.5‒8.0). Fig 3 shows a histogram for the total IES-R scores.

### Anxiety and depressive symptoms

The median anxiety score on the HADS was 4 (IQR = 1.17‒6.00), and the prevalence of anxiety was 16.6% (95% CI: 14.1‒19.4). The depression score of the HADS was 5 (IQR = 2‒8), and the prevalence of depression was 28.1% (95% CI: 25.1‒31.4). Fig 3 shows histograms of the total anxiety and depression scores.

### Co-occurrence of mental health-related symptoms

**Fig 4:**
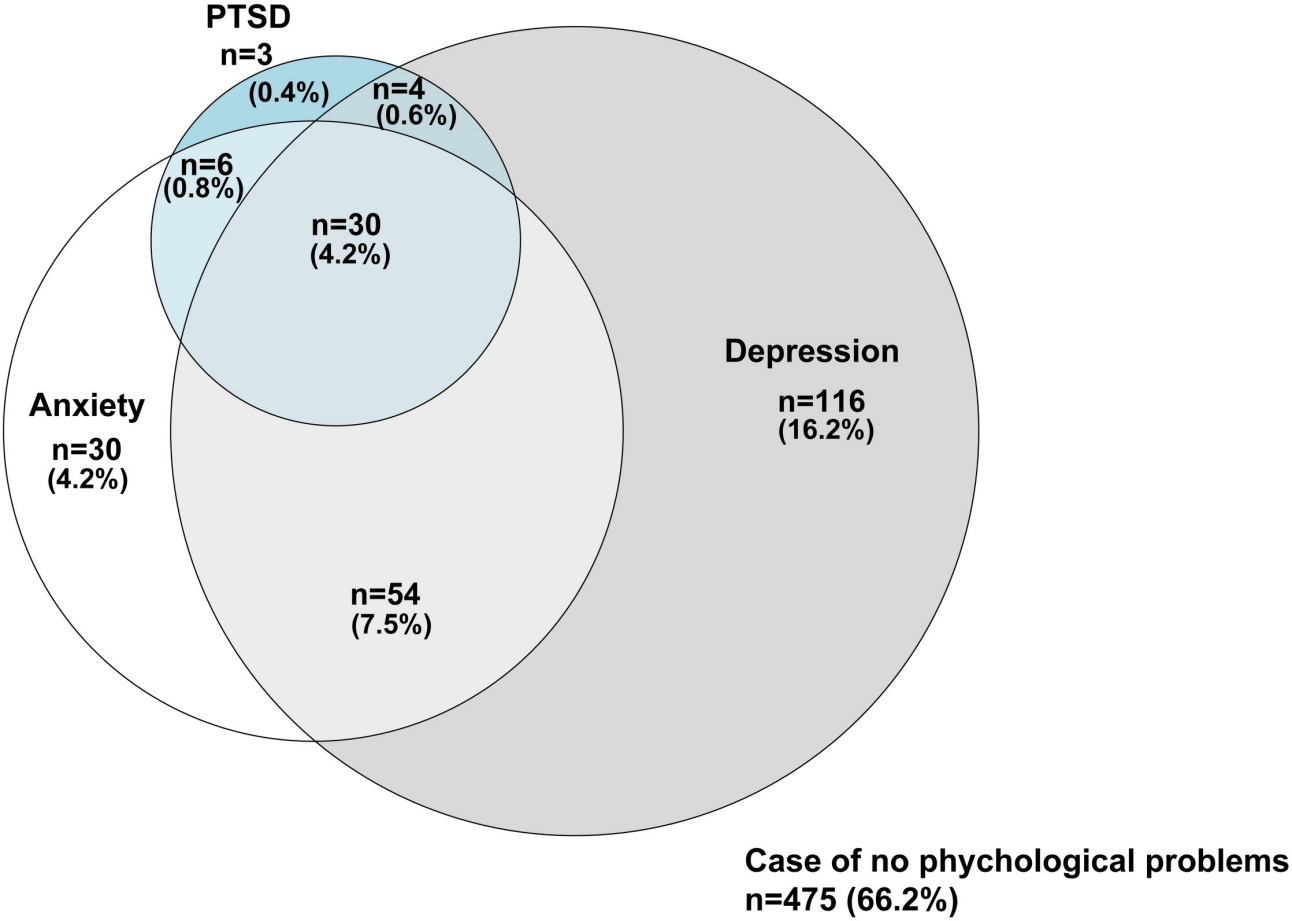
Venn diagram for PTSD, anxiety, and depression in patients living at home one year after discharge from the intensive care unit. PTSD, post-traumatic stress disorder.

The Venn diagram for PTSD, anxiety, and depression (based on the HADS) is shown in Fig 4. Two-hundred forty-three of 718 (33.8%) participants had at least one symptom, 30 of 43 participants (69.8%) with PTSD had both anxiety and depression, and 84 of 120 patients with anxiety (70.0%) had depression.

### Relationship of unscheduled admission with mental health

The subgroup analysis between elective surgery and unplanned admission is shown in S3 Table. The median total IES-R was higher for those with unscheduled admission as compared to elective surgery (4 [1–9] vs. 3 [1–8], p = .005, respectively). The results of the univariable and multivariable analysis using a multilevel linear regression model after multiple imputation for the association of variables with the severity of PTSD symptoms are shown in Table 2. The APACHE II score was non-significantly associated with the severity of PTSD symptoms in the univariable analysis. In a multivariable general linear model adjusted for pre-defined covariates, unscheduled admission (β = 0.174, 95% CI: 0.017–0.330, p = .029), psychiatric history (β = 0.741, 95% CI: 0.276–1.204, p = .002), and delirium (days) in the ICU (β = 0.021, 95% CI: 0.007–0.086, p = .021) were independent factors associated with PTSD severity (Table 2).

**Table 2:** Risk factors of the severity of post-traumatic stress disorder symptoms^a^ in univariable and multivariable analyses using a multilevel linear regression model.

Characteristic	Unadjusted		Adjusted	
	β (95% CI)^b^	p	β (95% CI)^b^	p
**Age**	-0.006 (-0.011 to -0.001)	.035	-0.005 (-0.011 to 0.001)	.064
**Male**	-0.119 (-0.289 to 0.052)	.173	-0.122 (-0.293 to 0.049)	.162
**MV days**	0.020 (0.001 to 0.040)	.048	-0.007 (-0.025 to 0.038)	.672
**ICU LOS**	0.008 (-0.006 to 0.023)	.247	-0.020 (-0.420 to 0.001)	.066
**Unscheduled admission**	0.201 (0.048 to 0.354)	.008	0.174 (0.017 to 0.330)	.029
**APACHE II**	0.004 (-0.007 to 0.015)	.449		
**Psychiatric history**	0.789 (0.323 to 1.256)	.001	0.741 (0.276 to 1.204)	.002
**Benzodiazepines**	0.386 (0.079 to 0.693)	.014	0.313 (-0.019 to 0.649)	.064
**Sepsis**	0.105 (-0.129 to 0.340)	.379		
**Trauma**	-0.133 (-0.572 to 0.306)	.552		
**Hospital LOS**	0.002 (0.000 to 0.005)	.045		
**Delirium days**	0.039 (0.010 to 0.069)	.008	0.021 (0.007 to 0.086)	.021
**Coma days**	0.022 (-0.005 to 0.047)	.104		

^a^Severity of post-traumatic stress symptom was from total Impact of Event Scale-Revised score.

^b^Because the Impact of Event Scale-Revised data were not normally distributed, we added + 1 to the sum of individual Impact of Event Scale-Revised scores (to avoid infinity upon transformation); then, we transformed them to the natural logarithm for analysis.

Note: These results were analyzed after multiple imputation.

MV, mechanical ventilation; ICU, intensive care unit; LOS, length of stay; APACHE II, Acute Physiology and Chronic Health Evaluation II.

Similarly, in the subgroup analysis (S3 Table), the median anxiety score was higher for those with unscheduled admission as compared to elective surgery (4 [2–7] vs. 3 [1–6], p = .019, respectively). The results of the univariable and multivariable analysis using a multilevel linear regression model for the association of variables with the severity of anxiety symptoms are shown in Table 3. In a multilevel linear model adjusted for pre-defined covariates, unscheduled admission (β = 0.174, 95% CI: 0.017 to 0.330, p = .029), psychiatric history (β = 0.547, 95% CI: 0.014 to 1.080, p = .044), and being male (β = -0.651, 95% CI: -1.221 to -0.080, p = .025) were independent factors associated with the severity of anxiety (Table 3).

**Table 3:** Risk factors of the severity of anxiety symptoms in univariable and multivariable analyses using a multilevel linear regression model.

Characteristic	Unadjusted		Adjusted	
	β (95% CI)	p	β (95% CI)	p
**Age**	-0.011 (-0.029 to 0.008)	.254	-0.010 (-0.028 to 0.009)	.320
**Male**	-0.644 (-1.214 to -0.075)	.026	-0.651 (-1.221 to -0.081)	.025
**MV days**	0.076 (0.009 to 0.142)	.026	0.026 (-0.063 to 0.115)	.567
**ICU LOS**	0.052 (0.004 to 0.100)	.034		
**Unscheduled admission**	0.584 (0.071 to 1.098)	.026	0.547 (0.014 to 1.080)	.044
**APACHE II**	0.007 (-0.031 to 0.044)	.731		
**Psychiatric history**	1.369 (-0.228 to 2.966)	.093	1.095 (-0.497 to 2.687)	.177
**Benzodiazepines**	0.411 (-0.631 to 1.454)	.439	-0.101 (-1.220 to 1.018)	.860
**Sepsis**	0.536 (-0.248 to 1.321)	.180		
**Trauma**	-0.467 (-1.931 to 0.997)	.532		
**Hospital LOS**	0.009 (0.000 to 0.017)	.031		
**Delirium days**	0.116 (0.016 to 0.215)	.022	0.073 (-0.053 to 0.199)	.257
**Coma days**	0.104 (0.012 to 0.200)	.027		
**Malignancy**	0.270 (-0.387 to 0.927)	.421	0.423 (-0.244 to 1.090)	.214

MV, mechanical ventilation; ICU, intensive care unit; LOS, length of stay; APACHE II, Acute Physiology and Chronic Health Evaluation II

Additionally, in the subgroup analysis (S3 Table), the median depression score was higher for those with unscheduled admission as compared to elective surgery (5 [3–8] vs. 5 [2–8], p = .034, respectively). The results of the univariable and multivariable analysis using a multilevel linear regression model for the association of variables with the severity of depressive symptoms are shown in Table 4. In a multilevel linear model adjusted for pre-defined covariates, unscheduled admission (β = 0.630, 95% CI: 0.047 to 1.213, p = .034) and delirium (β = 0.168, 95% CI: 0.030 to 0.306, p = 0.017) were independent factors associated with the severity of depression (Table 4).

**Table 4:** Risk factors for the severity of depressive symptoms^a^ in the univariable and multivariable analyses using a multilevel linear regression model.

Characteristic	Unadjusted		Adjusted	
	β (95% CI)	p	β (95% CI)	p
**Age**	-0.002 (-0.022 to 0.018)	.828	-0.002 (-0.023 to 0.018)	.835
**Male**	-0.368 (-0.989 to 0.253)	.245	-0.393 (-1.015 to 0.229)	.215
**MV days**	0.041 (-0.032 to 0.114)	.270	-0.060 (-0.157 to 0.038)	.230
**ICU LOS**	0.057 (0.005 to 0.109)	.032		
**Unscheduled admission**	0.686 (0.128 to 1.244)	.016	0.630 (0.047 to 1.213)	.034
**APACHE II**	0.025 (-.0151 to 0.066)	.220		
**Psychiatric history**	1.116 (-0.623 to 2.855)	.208	1.039 (-0.697 to 2.775)	.241
**Benzodiazepines**	2.131 (0.832 to 3.430)	.001	0.560 (-0.661 to 1.780)	.369
**Sepsis**	0.649 (-0.248 to 1.321)	.136		
**Trauma**	0.467 (-1.126 to 2.060)	.566		
**Hospital LOS**	0.011 (0.002 to 0.020)	.019		
**Delirium days**	0.149 (0.0415 to 0.257)	.007	0.168 (0.030 to 0.306)	.017
**Coma days**	0.133 (0.033 to 0.233)	.009		
**Malignancy**	0.115 (-0.601 to 0.830)	.754	0.271 (-0.459 to 1.000)	.467

MV, mechanical ventilation; ICU, intensive care unit; LOS, length of stay; APACHE II, Acute Physiology and Chronic Health Evaluation II

### Quality of life

The mean (standard deviation) QOL score was 0.79 (0.17) and the VAS for health was 73.0 (16.7). The mean and standard deviation of QOL scores in each age group and the Japanese norm, extracted from a previous study [31], are shown in Table 5. The mean QOL score in these patients was slightly lower than the norm in patients, except for 20–29-year-old women.

**Table 5:** Mean EuroQOL—5 Dimension index score, compared with the age- and sex-matched Japanese population norm.

Age group (years/sex)	n	Study value Mean (SD)	Japanese population norm Mean (SD)
≥ 70			
Male	271	0.802 (0.190)	0.866 (0.155)
Female	136	0.765 (0.219)	0.828 (0.202)
60–69			
Male	143	0.847 (0.167)	0.911 (0.158)
Female	30	0.753 (0.205)	0.899 (0.105)
50–59			
Male	65	0.824 (0.173)	0.936 (0.101)
Female	19	0.894 (0.130)	0.928 (0.092)
40–49			
Male	38	0.825 (0.189)	0.941 (0.088)
Female	15	0.804 (0.169)	0.914 (0.102)
30–39			
Male	11	0.936 (0.065)	0.950 (0.080)
Female	7	0.883 (0.238)	0.937 (0.089)
20–29			
Male	11	0.787 (0.129)	0.945 (0.102)
Female	4	1.000 (0)	0.950 (0.084)

SD, standard deviation

### Sensitivity analysis

When trauma patients were excluded, the prevalence of PTSD changed from 6.0% to 6.2% (95% CI: 4.6–8.2%), the prevalence of anxiety changed from 16.6% to 16.7% (95% CI: 14.1–19.7%), and the prevalence of depression remained unchanged (28.1%; 95% CI: 24.8–31.5%). The median total IES-R, anxiety, depression, EQ-5D-5L QOL, and EQ-5D-5L VAS scores were 4 (IQR = 1–9), 4 (IQR = 1.17–6.00), 5 (IQR = 2–8), 0.8603 (IQR = 0.7026–1.000), and 75 (IQR = 65–85), respectively.

## Discussion

In our study, the prevalence of PTSD in ICU patients after discharge, based on an IES-R score > 24 points, was 6.0%, which was significantly lower than that reported in a previous meta-analysis of ICU patients [7]. Similarly, the prevalence of anxiety was 16.6%, which was lower than the 19% pooled prevalence reported in a previous meta-analysis [38]. Depression was more common in these patients than anxiety, with a prevalence of 28.2% [37]. We also found an overlap of symptoms of PTSD, anxiety, and depression in patients. Of the 43 patients with PTSD, three patients had only PTSD and 30 patients had both depression and anxiety. Owing to these conditions, the QOL of the Japanese ICU population was lower than that of an age- and sex-matched population norm one-year post-ICU discharge. A sensitivity analysis indicated that the prevalence of mental health symptoms and QOL were robust, and our findings were non-significantly affected by trauma patients.

There are some possible explanations for differences in the findings of PTSD prevalence between this study and the previous meta-analysis of ICU patients [7]. In the present study, patients’ diverse characteristics could have contributed to a lower prevalence of PTSD. Unplanned admission was an independent predictor of more severe PTSD, anxiety, and depressive symptoms after adjusting for covariates. Approximately half the patients were in the ICU for at least three nights after elective surgery. Based on the Japanese intensive care patients’ database registration [39], the proportion of the ICU population admitted to the ICU for planned surgery in Japan was greater than that in Australia and New Zealand [40], the United Kingdom [41], and Brazil [42]. Moreover, our study included only ICU survivors living at home, which probably led to an increase in the proportion of elective surgery patients. A previous study showed that anxiety was significantly lower in patients undergoing elective surgery than in those undergoing unplanned surgery [43]. Patients with unplanned admission to the ICU do not have the opportunity to psychologically prepare and often require more invasive emergency procedures (e.g., emergency tracheal intubation). This may have contributed to our findings. Additionally, this difference in populations could also have contributed to the higher prevalence of PTSD in a recent Japanese ICU study, which analyzed only emergency-admitted patients [10].

The older age of the population in this study may also have contributed to the lower prevalence of PTSD; although, some previous studies suggested that a younger age was associated with a lower prevalence of PTSD [17, 44]. The population of our study was older than the age of patients in a previous systematic review. In fact, 33% of the population in Japan is aged ≥ 60 years, and Japan had the oldest society worldwide in 2017 [45]. A recent meta-analysis suggested that older age was associated with the reduced PTSD development [19]. Thus, the older age of the patients in this study may have contributed to our findings concerning PTSD.

Nevertheless, a few recent studies indicated that the prevalence of PTSD may be relatively low in South or East Asian countries. Surprisingly, an Indian study indicated a 0% PTSD prevalence (by 180 days after ICU discharge) in 322 patients who had stayed in the ICU for more than 24 hours [44]. Similarly, a study conducted in Hong Kong reported that only 3.7% of patients receiving intensive care had PTSD one week post-discharge [9]. A World Mental Health survey reported that the prevalence of PTSD in Japan was lowest in the world [8]. Ethno-cultural factors may contribute to the prevalence of PTSD [46]; therefore, this factor may be associated with our findings. To clarify the epidemiology of PICS, further studies in East Asian countries are needed.

Mental illness severity may not have contributed to this difference. Hatch and colleagues [6] investigated the prevalence of PTSD using the PTSD Checklist‒Civilian Version at 1 year post-ICU discharge. They examined 3,151 patients who stayed in the ICU for more than 24 hours and revealed an 18% PTSD prevalence, which was significantly higher than our rate. However, their median APACHE score was 15 (11‒19), which was comparable to that in our study population. A recent systematic review also suggested that illness severity was not associated with the development of PTSD [19]; thus, it is unlikely that mental illness severity at admission contributed to the difference in the prevalence of PTSD between our study and previous studies.

The prevalence of depression (based on the HADS) in this study (28.2%) was comparable to that reported in a previous meta-analysis, which reported a prevalence of depression of 29% (23‒34%) at 12 or 14 months post-ICU discharge [37]. In contrast, another meta-analysis [38] showed that the pooled prevalence of anxiety (based on the HADS) was 34% (25‒42%) at 12 or 14 months post-ICU discharge, which was higher than our findings (16.6%). Risk factors for anxiety remain unclear; thus, we cannot explain the lower anxiety symptoms in the present study. Because previous studies have indicated that the severity of anxiety and PTSD symptoms were moderately correlated [47, 48], it may be that the lower rate of anxiety in our study was because of the same reasons as the lower prevalence of PTSD.

The QOL score of patients at one year post-ICU discharge was slightly lower in the present study than in the Japanese population. Similarly, a recent meta-analysis reported that patients had a lower QOL after a critical illness as compared to age- and sex-matched populations [49]. We did not measure pre-ICU QOL; thus, we do not know if the slightly lower QOL was ICU-related. Further studies that assess QOL before and after ICU stay are required.

In this large multicenter study, we clarified the prevalence of PICS in a Japanese population living at home after ICU discharge. No multicenter data had been reported for Japan previously. With a high response rate, the population in this study, derived from 12 centers across Japan, is representative of the Japanese ICU population. We found that the prevalence of PTSD and anxiety in Japanese patients one year after discharge from an ICU was relatively low. Nevertheless, unscheduled admission was an independent predictor for more severe PTSD symptoms.

This study had some limitations. First, we collected data related to participants’ hospital stays retrospectively. Psychiatric history, which is significantly associated with post-ICU mental health, was obtained from medical charts; thus, patients’ problems may have been underestimated. Additionally, data that may be associated with mental health were lacking, including factors such as education level [50] and social support [51], which were shown to be associated with PTSD after intensive care. A prospective study is warranted to explore the risk of PTSD in this population.

Second, 179 patients could not be contacted (8.4% of screened patients). We excluded these patients, and it was not possible to determine whether these patients were deceased or unable to complete the questionnaire. We speculate that the major reason for being unable to contact these patients was because they died, were admitted to a healthcare facility, were incapacitated, or because they did not have enough time to respond, rather than a reason related to their mental status or QOL. Thus, this limitation is not likely to have meaningfully contributed to our findings.

Third, it is possible that patients who refused to participate and the non-responders might have had PTSD symptoms, given that avoidance is a symptom of PTSD. However, a previous randomized controlled trial conducted with a post-ICU population compared performing a QOL scale assessment alone to performing both the HADS and PTSD screening in addition to QOL scale assessments [52]. They found that the response rates to the questionnaires were non-significantly different between groups; thus, we posit that this issue did not impact our findings.

Fourth, we excluded patients with disorders or trauma of the central nervous system because we thought it was not clear whether the self-completion survey form could be filled out accurately in those population. A previous study [53] indicated that the prevalence of PTSD in patients with traumatic brain injury was 18.6% at one year post-injury. Additionally, another study [54] reported that 25.5% of patients had PTSD three years after subarachnoid hemorrhage. Thus, our findings of prevalence may be underestimated and should not be generalized for all ICU populations.

This study population was mixed, including medical, surgical, and small population of trauma patients; however, it reflected the general Japanese ICU situation excluding patients with central nervous system disorders or injuries. This study could be the largest epidemiological investigation of the general ICU population in Japan. Further studies are needed to clarify if PICS is more prevalent in specific disorders, such as sepsis.

Definitive preventive measures to offset PICS have not established, specifically concerning the prevention of and treatment for mental illness. A recent meta-analysis showed that ICU diaries [55] effectively reduced depression; however, another recent meta-analysis [56] suggested that ICU diaries did not reduce anxiety or PTSD. Moreover, nurse-led psychological interventions during patients’ ICU stay [57] and nurse-led intensive care recovery program after patients’ ICU stay [58] failed to prevent PTSD. Therefore, recently, multidisciplinary and multimodality interventions have been emphasized [12]. When healthcare providers have limited medical resources, our findings may contribute to the detection of high-risk patients. Following-up with unplanned admission patients and considering multidisciplinary and multimodality interventions are critical.

## Conclusions

The results of this multicenter study indicated that approximately one-third of all patients experienced psychological problems after ICU discharge. Screening procedures during hospital stay and systems for adequate follow-up must be developed. Moreover, unscheduled admission to the ICU was predictive of more severe symptoms of PTSD, anxiety, and depression; thus, this population requires closer attention and careful follow-up.

## Supporting information

S1 Table Characteristics, delirium-screening method, number of analyzed participants, and implications for intensive care unit diary by institution. (DOCX)

S2 Table Details of missing items for the IES-R and HADS. (DOCX)

S3 Table Subgroup analysis between elective surgery and unplanned admission. (DOCX)

S1 Text The 12 intensive care units that participated in this study. (DOCX)

S1 Data The Data for histograms of IES-R and HADS. (XLSX)
