# Supplementary material for: Prevalence of and risk factors for post-intensive care syndrome: Multicenter study of patients living at home after treatment in 12 Japanese intensive care units, SMAP-HoPe study
Source: PLoS One. 2021 May 27;16(5):e0252167. doi: 10.1371/journal.pone.0252167 (PMC8158919; doi:10.1371/journal.pone.0252167)
Supplement: S1 Table — (DOCX) [file pone.0252167.s001.docx]

S1 Table. Characteristics, delirium-screening method, number of analyzed subjects, and implication for intensive care unit diary by institution

|  | Center | | | | | | | | | | | | |
| --- | --- | --- | --- | --- | --- | --- | --- | --- | --- | --- | --- | --- | --- |
| Hospital type | Private | University | University | Private | University | Public | University | University | Public | University | University | University |  |
| Type of ICU | med-surg | med-surg | med-surg | CV surgery | med-surg and emergency | med-surg | med-surg | med-surg | med-surg | med-surg | med-surg | med-surg |  |
| No. of ICU beds | 8 | 16 | 8 | 12 | 16 | 8 | 14 | 6 | 16 | 6 | 10 | 12 |  |
| Study participants, n (%) | 53 (7.0) | 56 (7.4) | 53 (7.0) | 54 (7.2) | 60 (8.0) | 32 (4.2) | 102 (13.5) | 86 (11.4) | 112 (14.9) | 18 (2.4) | 61 (8.1) | 67 (8.9) |  |
| Duration for enrollment | 10 months | 7 months | 9 months | 10 months | 9 months | 9 months | 10 months | 10 months | 9 months | 10 months | 10 months | 9 months |  |
| Type of delirium assessment scale | CAM-ICU | CAM-ICU | ICDSC  and  CAM-ICU | CAM-ICU | ICDSC | ICDSC | ICDSC  and  CAM-ICU | ICDSC | CAM-ICU | ICDSC | CAM-ICU | CAM-ICU |  |
| Number of delirium assessments a day | 2 | 3 | 3 | 4~5 | 6 | 3 | 2~5 | 3 | 3 | 3 | 4 | 6 |  |
| Use of ICU-diary | Never | Never | Sometimes | Never | Rarely | Rarely | Occasionally | Often | Rarely | Rarely | Rarely | Never |  |

CV surgery, cardiovascular surgery; Med-Surg, medical-surgical; ICU, intensive care unit; CAM-ICU, Confused Assessment Method for the Intensive Care Unit; ICDSC, Intensive Care Delirium Screening Checklist.

For cardiac patients, elective critical care consultation; For others, mandatory critical care consultation

ICU organization model was referred
