# Supplementary material for: Prevalence of and risk factors for post-intensive care syndrome: Multicenter study of patients living at home after treatment in 12 Japanese intensive care units, SMAP-HoPe study
Source: PLoS One. 2021 May 27;16(5):e0252167. doi: 10.1371/journal.pone.0252167 (PMC8158919; doi:10.1371/journal.pone.0252167)
Supplement: S2 Table — (DOCX) [file pone.0252167.s002.docx]

S2 Table. Details of Missing Item of IES-R and HADS.

IES-R

| Number of Missing Items | 1 | 2 | 4 | 6 | 7 | 8 | 9 | 14 | 15 | 18 | 21 | 22 | Total |
| --- | --- | --- | --- | --- | --- | --- | --- | --- | --- | --- | --- | --- | --- |
| Number of Subjects | 17 | 3 | 1 | 2 | 1 | 1 | 1 | 12 | 1 | 1 | 3 | 18 | 61 |
| Percentage of total missing subjects | 27.8 | 4.9 | 1.7 | 3.2 | 1.6 | 1.6 | 1.6 | 19.7 | 1.6 | 1.6 | 4.9 | 29.5 | 100 |

IES-R

Remaining data after imputation using “half rule”

| Number of Missing Items | 9 | 14 | 15 | 18 | 21 | 22 | Total |
| --- | --- | --- | --- | --- | --- | --- | --- |
| Number of Subjects | 1 | 12 | 1 | 1 | 3 | 18 | 36 |

Details of Missing Items of HADS

Raw data

| Number of Missing Items | 1 | 2 | 3 | 4 | Total |
| --- | --- | --- | --- | --- | --- |
| Number of Subjects | 54 | 4 | 5 | 3 | 66 |
| % | 7.16 | 0.53 | 0.66 | 0.40 | 8.75 |

IES-R, Impact of Event Scale‒revised; HADS, Hospital Anxiety and Depression Scale
