# Supplementary material for: Prevalence of and risk factors for post-intensive care syndrome: Multicenter study of patients living at home after treatment in 12 Japanese intensive care units, SMAP-HoPe study
Source: PLoS One. 2021 May 27;16(5):e0252167. doi: 10.1371/journal.pone.0252167 (PMC8158919; doi:10.1371/journal.pone.0252167)
Supplement: S3 Table — (DOCX) [file pone.0252167.s003.docx]

S3 Table. Subgroup analysis between elective surgery and unplanned admission.

|  |  | Elective surgery, n=356 | Unplanned admission, n=398 | p-value |
| --- | --- | --- | --- | --- |
| Age, median [IQR] |  | 70.0 [61.0-78.0] | 70.0 [49.5-79.0] | 0.43 |
| Female, n (%) |  | 104 (29.2) | 109 (27.4) | 0.627 |
| Reason for ICU admission, n (%) | | | |  |
|  | CV surgery | 250 (70.2) | 56 (14.1) | 0.37 |
|  | CHF/AMI/Arrhy | 0 (0.0) | 122 (30.7) |  |
|  | Sepsis | 0 (0.0) | 78 (19.6) |  |
|  | Abdominal surgery | 54 (15.2) | 13 (3.3) |  |
|  | ENT surgery | 30 (8.4) | 1 (0.3) |  |
|  | Respiratory Failure | 0 (0.0) | 30 (7.5) |  |
|  | Aortic Dissection (non-operative) | 0 (0.0) | 26 (6.5) |  |
|  | Other surgery | 22 (6.2) | 3 (0.8) |  |
|  | Trauma | 0 (0.0) | 23 (5.8) |  |
|  | Others | 0 (0.0) | 46 (11.6) |  |
| Surgery, n (%) | | 356 (100.0) | 109 (27.4) | <0.001 |
| APACHE II, median [IQR] | | 14.00 [10.00, 19.25] | 15.00 [11.00, 20.00] | 0.076 |
| MV use, n (%) | | 292 (82.0) | 224 (56.3) | <0.001 |
| MV days, median [IQR] | | 2.00 [1.00, 2.00] | 2.00 [0.00, 4.00] | 0.666 |
| Benzazepines, median [IQR] | | 15 (4.2) | 34 (8.5) | 0.018 |
| Psychological history, n (%) | | 9 (2.5) | 11 (2.8) | 1 |
| Delirium during ICU stay, n (%) | | 45 (12.6) | 128 (32.2) | <0.001 |
| Delirium days, median [IQR] | | 0.00 [0.00, 1.00] | 0.00 [0.00, 2.00] | <0.001 |
| ICU LOS, median [IQR] | | 5.00 [4.00, 6.00] | 6.00 [5.00, 9.00] | <0.001 |
| Hospital LOS, median [IQR] | | 29.00 [22.00, 43.00] | 25.50 [16.25, 40.00] | <0.001 |
| Anxiety Score, median [IQR] | | 3.00 [1.00, 6.00] | 4.00 [2.00, 7.00] | 0.019 |
| Anxiety, n (%) | | 45 (12.6) | 80 (20.1) | 0.006 |
| Depression Score, median [IQR] | | 5.00 [2.00, 8.00] | 5.00 [3.00, 8.00] | 0.034 |
| Depression, n (%) | | 92 (25.8) | 120 (30.2) | 0.195 |
| Total IES-R score, median [IQR] | | 3.00 [1.00, 8.00] | 4.00 [1.00, 9.00] | 0.005 |
| PTSD, n (%) | | 17 (5.0) | 26 (6.9) | 0.345 |
| QOL score, median [IQR] | | 0.87 [0.74, 1.00] | 0.84 [0.70, 1.00] | 0.287 |
| EQ-5D-5L VAS, median [IQR] | | 75.00 [70.00, 85.00] | 75.00 [60.00, 85.00] | 0.067 |

IQR, interquartile range; ICU, intensive care unit; CV, cardiovascular; CHF/AMI/Arrhy, Congestive Heart Failure/Acute Myocardial Infarction/Arrhythmia; ENT, ear nose threat; APACHE II, acute physiology and chronic health evaluation II; MV, mechanical ventilation; ICU, intensive care unit; LOS, length of stay; IES-R, impact of event scale revised; QOL, quality of life; VAS, visual analog scale; EQ-5D-5L, Euro-quality of life questionnaire; PSTD, post-traumatic stress disorder.
