# Supplementary material for: Prevalence of and risk factors for post-intensive care syndrome: Multicenter study of patients living at home after treatment in 12 Japanese intensive care units, SMAP-HoPe study
Source: PLoS One. 2021 May 27;16(5):e0252167. doi: 10.1371/journal.pone.0252167 (PMC8158919; doi:10.1371/journal.pone.0252167)
Supplement: S1 Text — (DOCX) [file pone.0252167.s004.docx]

S1 Text

**The ICU participated in this study**

Emergency and Critical Care Medical Center, Osaka City General Hospital, Osaka, Osaka, Japan

Intensive Care Unit, Nara Medical University Hospital, Kashihara, Nara, Japan

Intensive Care Unit, Nippon Medical School Musashikosugi Hospital, , Kawasaki, Kanagawa, Japan

Intensive Care Unit, University of Tsukuba Hospital, Tsukuba, Ibaraki, Japan

Intensive Care Unit, Tohoku Medical and Pharmaceutical University Hospital, Sendai, Miyagi, Japan

Intensive Care Unit of Advanced Emergency Medical Service Center, Japanese Red Cross Maebashi Hospital, Maebahi, Gunma, Japan

Intensive Care Unit, Jichi Medical University Hospital, Shimotsuke, Tochigi, Japan

Intensive Care Unit, Sakakibara Heart Institute, Fuchu, Tokyo, Japan

Heart Center, Kanazawa Medical University Hospital, Kahoku, Ishikawa, Japan

Intensive Care Unit & Cardiac Care Unit, Japanese Red Cross Fukuoka Hospital, , Fukuoka, Fukuoka, Japan

Intensive Care Unit, Sapporo Medical University Hospital, Sapporo, Hokkaido, Japan

Intensive Care Unit, Naha City Hospital, Naha, Okinawa, Japan
